# Monitoring regulatory T cells as a prognostic marker in lung transplantation

**DOI:** 10.3389/fimmu.2023.1235889

**Published:** 2023-09-25

**Authors:** Mohammad Afzal Khan, Christine L. Lau, Alexander Sasha Krupnick

**Affiliations:** School of Medicine, University of Maryland, Baltimore, MD, United States

**Keywords:** regulatory T cell, chronic lung allograft dysfunction, bronchiolitis obliterans syndrome, immune tolerance, tissue repair

## Abstract

Lung transplantation is the major surgical procedure, which restores normal lung functioning and provides years of life for patients suffering from major lung diseases. Lung transplant recipients are at high risk of primary graft dysfunction, and chronic lung allograft dysfunction (CLAD) in the form of bronchiolitis obliterative syndrome (BOS). Regulatory T cell (Treg) suppresses effector cells and clinical studies have demonstrated that Treg levels are altered in transplanted lung during BOS progression as compared to normal lung. Here, we discuss levels of Tregs/FOXP3 gene expression as a crucial prognostic biomarker of lung functions during CLAD progression in clinical lung transplant recipients. The review will also discuss Treg mediated immune tolerance, tissue repair, and therapeutic strategies for achieving *in-vivo* Treg expansion, which will be a potential therapeutic option to reduce inflammation-mediated graft injuries, taper the toxic side effects of ongoing immunosuppressants, and improve lung transplant survival rates.

## Introduction

**Figure 1:**
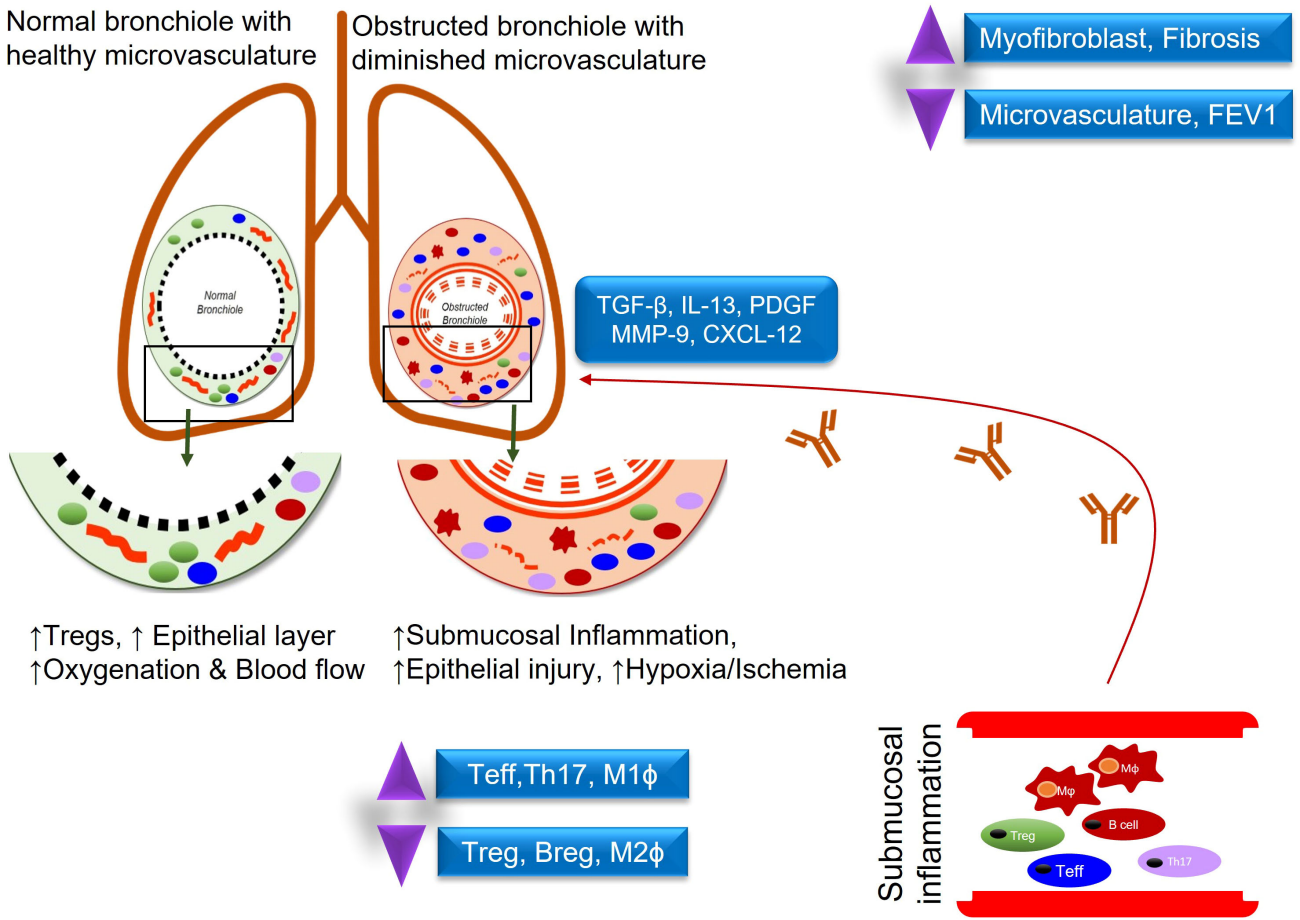
Demonstrates an inflammatory response in normal vs obstructed bronchioles after lung transplantation.

Lung transplantation is a life-saving surgical procedure for patients with end-stage lung diseases. Unfortunately, this treatment strategy is limited by the occurrence of CLAD which occurs when the patient’s immune system relentlessly attacks the transplanted organ, disrupts the microvascular flow, and ultimately leads to irreversible small airway fibrosis. CLAD is a major cause of mortality in the first ten years and there are no current immunosuppressive regimens that can sufficiently rescue the restoration of functional microvascular flow during rejection (**Figure 1**) (1, 2). For recipients, lung transplantation has led to improved quality of life and longevity but outcomes among transplant recipients are quite heterogeneous with under 60% transplant survival at 5 years and under 20% transplant survival at 10 years post-transplantation (3, 4). This concept of regulation is likely not the result of actions of a particular cellular subset, but rather the collective effect of signaling between Tregs, antigen presenting cells (APC), and metabolites which have additional regulatory logistics such as the release of IL-10 or TGF-β, and a balance between Th17(IL-6, CXCL10) –Tregs (CCL22, IL-10) may foresee the risk of CLAD progression (5, 6). Once the mechanisms underlying regulation are better delineated, perhaps these processes can be augmented in all lung transplant recipients as part of a broader novel immunologic approach to transplantation.

## Immunotolerance phase

The immune system guards the host against a broad range of foreign pathogenic microorganisms and tissue antigens, which involve an organized display of cellular and molecular interactions to counter-attack foreign entities through discrete recognition of antigenic peptides and thereby establish a powerful effector response, and long-term immunologic memory (7). However, this effector response remains tightly regulated, but critical tissue injuries and organ malfunctions of the host may develop during abnormal immune reactions, which involve autoimmunity, hyper-responsiveness, and organ rejections (8–12). Although, this is a very critical issue and a major challenge to drug discovery programs to establish a constant phase of immunological tolerance to avoid injuries to the host tissues, and thus key cellular and molecular signaling of immunological self-tolerance will highlight the crucial immune checkpoints to regulate powerful immune responses to transplant recipients. Treg is one of the essential immune cells, which suppress immune responses, maintain self-immunotolerance, and contribute vitally to tissue and vascular repair (13, 14). Treg can inhibit the proliferation of T cells via direct cell-cell contact, through granzyme B and perforin-mediated; or through reducing costimulatory signals and inhibiting antigen presentation (15, 16). Tregs routinely play a major role in maintaining immunological tolerance to self-antigens and suppress immune responses injurious to the transplant recipients (17–20).

**Figure 2:**
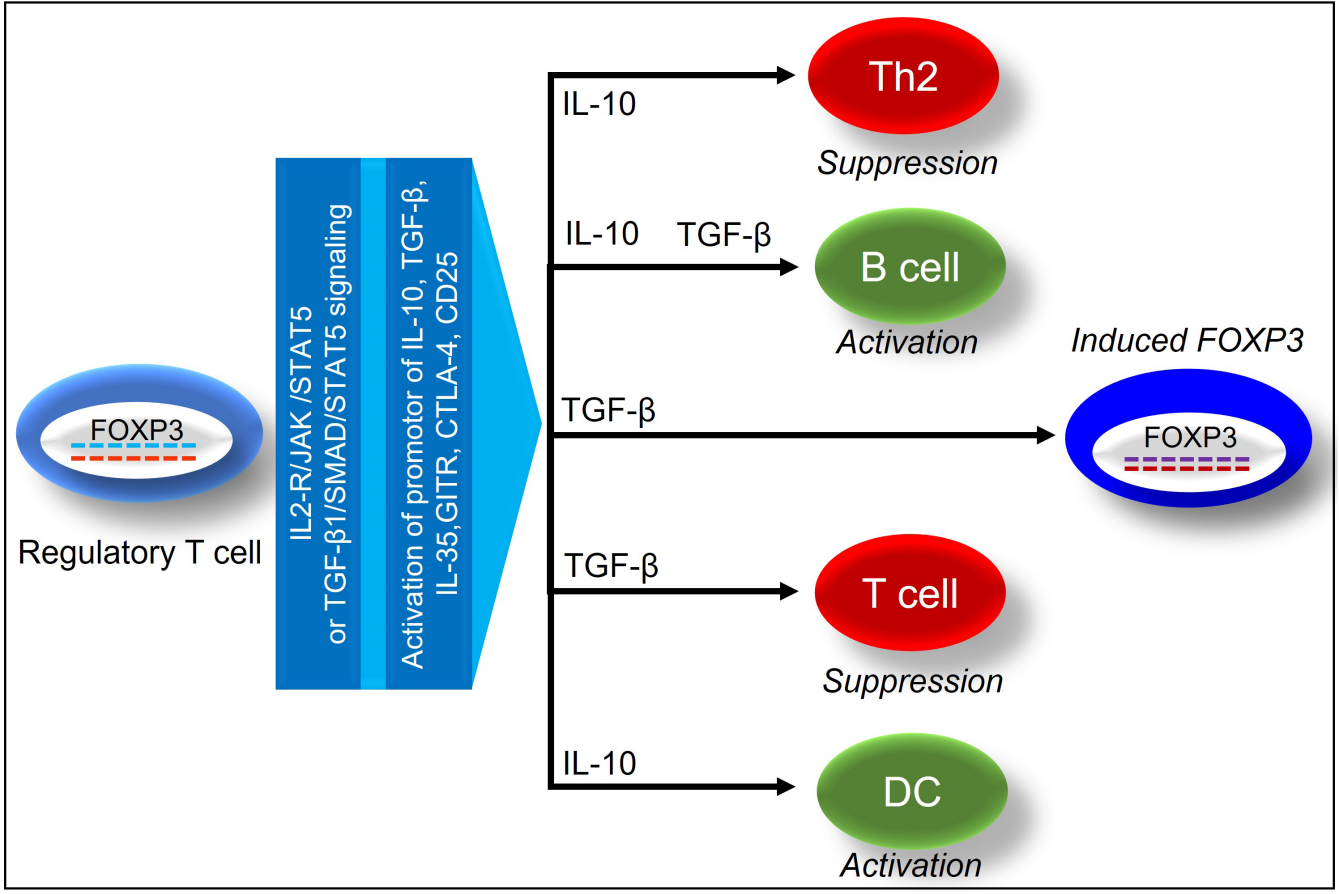
Demonstrates the immunosuppressive effects of FOXP3^+^ regulatory T cells on tissue and vascular repair.

Functionally mature T cell subsets-Tregs in the thymus are a unique CD4^+^ T-cell subpopulation, which in mice is characterized by the surface expression of CD25, nuclear expression of FOXP3, and secrete IL-10, TGF-β to suppress heightened immune responses, and also trigger inducible Treg expansion (21–23). Unlike mouse, Human Treg population is highly heterogeneous, and different markers including CD3, CD4, CD25, and FOXP3 have been minimally required to define human Treg cells (24). Besides, staining for Ki67 and CD45RA showed to provide additional information on the activation status of Tregs (25). Demethylation of FOXP3 determines stable FOXP3 expression in clinical transplants, which has been widely recognized as an essential transcription factor in Tregs (26, 27). During an alloimmune inflammation, Hypoxia Inducible Factor 1 Subunit Alpha (HIF-1α) expression upregulates Th17 cells while downregulates Tregs through the binding to FOXP3 (28, 29). IL-2 plays an important role in stabilizing FOXP3 gene expression, and a high expression of the IL-2 receptor correspond dictate the effective immunosuppressive functions of Tregs (30). In response to IL-2 receptor signaling, Janus kinases (JAKs) initiate phosphorylation of Signal transducer and activator of transcription 5 (STAT5) and an activated STAT5 binds to the FOXP3 promoter and conserved non-coding sequence 2 (CNS2), signaling Treg activation (31). In addition, IL-6 induces CNS2 methylation to suppress FOXP3 expression, and IL-21 activates STAT3 to suppress FOXP3 expression, whereas TNF-a dephosphorylates & restores Treg function (32, 33) (**Figure 2**).

## Immunosuppression

An immunosuppressive regimen remains essential for lung transplantation success, and a wide variety of immunosuppressive agents, as well as combinations of them, are available for use after lung transplantation, giving patients more personal choice (34, 35). Although these drugs are effective, their side effects can be severe, reducing a patient’s life expectancy. Consequently, new immunosuppressive therapies are required that promote immune tolerance without the side effects currently observed. An effective immunosuppression can be achieved by combining various signaling pathways that work through the immunomodulation functions of various immune cells, and the selective inhibition of effector and memory T cells through these pathways could theoretically be used to decrease the amount of immunosuppressive drugs and promote the induction of tolerance (36). In addition, Treg-based immunomodulation may reduce the toxic effects associated with current immunosuppressive treatments (37–39). The utilization of this approach could be a game-changer when it comes to managing transplanted patients, improving outcomes, and reducing toxic treatments. The current immunosuppressive agents used in clinics modulate Treg activity through a variety of signaling pathways (40). Such agents are effective in controlling inflammatory conditions; however, their use is associated with several adverse effects. An immunosuppressive drug commonly used in transplantation is calcineurin inhibitors (CNIs), mammalian Target of Rapamycin inhibitors (mTOR), corticosteroids, mycophenolate preparations, anti-thymocyte globulin (ATG), anti-CD25 antibody, anti-CD52 antibody, Lymphocyte function-associated antigen-3 (LFA-3) fusion protein antibody, anti-IL-6R antibody, anti-CD28 antibody, and Cytotoxic T-lymphocyte-associated protein 4 (CTLA4) antibody (36, 41, 42). In general, immunosuppression affects immune cells of the graft, thereby playing a crucial role in tissue repair, fibrosis progression, and lung function following lung transplantation (**Table 1**).

**Table 1:** Various Immunosuppressants drugs and Treg levels during transplantation.

	Immunosuppressants	Target pathways	Tregs
1	Calcineurin inhibitors	↓ Calcineurin & NFAT, FOXP3	↓
	CTLA4-Ig	Blocks CD28 signaling	↓
	anti-IL-2R	Blocks IL-2 receptor signaling	↓
2	m*TOR* inhibitors	↓ mTORc1	↑
	histone deacetylases inhibitor	↓ Histone deacetylases and 1FOXP3	↑
	Low-dose IL-2	↑ IL-2R signaling on Tregs.	↑
	Rabbit anti-thymocyte globulin	T cells markers.	↑
	Anti-IL-6R	prevents IL-6/IL-6R binding	↑
3	Complement inhibitors	Blocks C5 cleavage	-
	Steroids	↓ pro-inflammatory cytokines	-
	Antiproliferative agents	Inhibit purine synthesis	-

↓ (Downregulation); ↑ Upregulation; - (No effects).

## Tregs and tissue repair

The cytokines and growth factors play important roles in cell proliferation, migration, and matrix synthesis, which make them critical to fundamental homeostatic and pathophysiological processes such as wound healing, inflammation, tissue repair and fibrosis (43). Depending on the cytokine and its role, it may be appropriate to either enhance (recombinant cytokine, gene transfer) or inhibit (cytokine or receptor antibodies, soluble receptors, signal transduction inhibitors, antisense) the cytokine to achieve the desired outcome. Consequently, cytokines, which are central to this constellation of events for coordinating multiple cell types, have become targets for therapeutic intervention to modulate the wound healing process, which is crucial to transplant survival. In wound healing, several immune cells participate in the process, including platelets, neutrophils, macrophages, fibroblasts, lymphocytes, epithelial and endothelial cells. However, Tregs, as well as their associated regulatory mediators, help to protect the tissue from inflammation (44–48). During hemostasis, platelets release transforming growth factor-β1 (TGF-β1), Platelet-derived growth factor (PDGF), fibroblast growth factor (FGF-2), and Vascular endothelial growth factor (VEGF) to recruit neutrophils and macrophages, while neutrophils release reactive oxygen species (ROS), nitric oxide (NO), proteases, VEGF, and IL-17 to destroy pathogens (49–51). Besides, NK cells secrete IFN-γ, TNF-α and also release perforins and granzymes that are cytotoxic to infected cells (52). Moreover, neutrophils release TNF-α, IL-1β, IL-6, and MCP-1, which attract monocytes and dendritic cells and activate T cells that cause Th1 pro-inflammatory responses (53). In the inflammatory phase of acute wound healing, macrophages secrete IL-1, VEGF, FGF-2, TNF- α, IL-6, IFN-γ, TGF-β, and PDGF, which promote the proliferation of fibroblasts, keratinocytes, and epithelial cells, whereas in the remodeling phase IL-4, IL-10, and IL-13 induce the transition of M1 to M2 macrophages (50, 53). Besides, other cells, such as mesenchymal stem cells (MSCs) and fibroblasts, secrete Tumor necrosis factor- (TNF) stimulated gene-6 (TSG-6), which promotes wound healing by limiting macrophage activation, inflammation, and fibrosis (54, 55). M2 macrophages generally inhibit inflammation and promote tissue repair through IL-10 and TGF-β, which stimulate extra cellular matrix (ECM) synthesis, angiogenesis, and fibroblast proliferation (44). During inflammation, lymphocytes are also recruited to the wound and release IFN- γ, TGF-β, IL-10, IL-2, IL-17, and IL-22 (56). Later, angiogenesis replaces damaged vessels with granulation tissue, in which epidermal cells, fibroblasts, vascular endothelial cells, and macrophages produce β-FGF, TGF-β, and VEGF to bolster angiogenesis (57). VEGF induces angiogenesis through adenosine, which in turn stimulates hypoxia-induced proliferation, therefore A_2A_ receptors, is now considered a potent regulator of the early stages of tissue repair caused due to overactivation of various inflammatory mediators (9, 58–60). Tregs promote tissue repair through various regulatory cytokines, which include IL-10, TGF-β, IL-33, IL-35 and amphiregulin (61–63) (**Figure 2**). IL-10, an anti-inflammatory cytokine, favors tissue repair, and regulate FOXP3 (64). IL-10 is a potent antifibrotic, reparative, as well as vasculo-protective cytokine that assists in the repair of tissue following a sporadic alloimmune response during transplantation (46, 65–71). The anti-inflammatory properties of IL-10 help to suppress the production of pro-inflammatory cytokines such as IFN- γ, IL-2, IL-3, and TNF- α by Th1 cells, mast cells, NK cells, endothelial cells, eosinophils, and macrophages (72–78).

In addition to limiting collateral tissue damage caused by uncontrolled immune responses, IL-10 helps maintain the regulatory microenvironment by upregulating TSG-6, M2 macrophages, and, tolerogenic dendritic cells (DC-10), antigen-specific T regulatory type 1 (Tr1), while suppressing Th1/Th17 effector immunity (65, 67, 68, 71, 73, 79, 80). Through the surface expression of TSG-6, FOXJ1, Fascin-1, and β-catenin proteins, IL-10 enhances microvascular supply, tissue oxygenation, and airway epithelium regeneration in allografts, further supporting the therapeutic benefits during wound healing and tissue repair (46, 65, 66, 81). The relationship between inflammation and fibrogenesis has led to IL-10 being identified as a potential antifibrotic target as well as a gatekeeper of fibrotic/antifibrotic signaling, so immune and cell-based therapies aiming to capitalize on IL-10 as a target could be effective in treating lung transplanted patients suffering from delayed would healing. These studies supported that IL-10 is vital for regenerative functions, and associated with a proportional increase in another anti-inflammatory protein TSG-6, and further upregulation of CD4^+^FOXP3^+^ Tregs, which thereby support the reestablishment of microvascular supply, tissue oxygenation, airway epithelial repair, and suppression of collagen deposition in allografts (17, 19, 46, 64, 82, 83). TSG-6 has been established to regulate pro-inflammatory cytokines and augment tissue repair in various animal models (84, 85) while suppressing inflammatory reactions triggered by ischemia in the heart and thereby limiting the destruction of cardiomyocytes (86). However, TSG-6 gene inactivation has been associated with the upregulation of inflammatory immune response, while over-expression of the TSG-6 gene has been associated with the downregulation of inflammatory responses (87–92). TSG-6 is a crucial regulatory mediator secreted by fibroblasts, monocytes, and mesenchymal stem cells to facilitate healing in an inflamed or metabolically active tissue microenvironment (92). TSG-6 is rapidly upregulated in response to inflammatory cytokines to protect tissues from inflammation, and is also involved in anti-inflammatory, antifibrotic, proangiogenic, and analgesic functions during inflammation (93). TSG-6 can modulate matrix structure and organization by upregulating several regulatory cells, such as Tregs, M2 macrophages, Matrix metalloproteinases (MMPs), and associated anti-inflammatory cytokines, thereby suppressing proinflammatory cytokines (IL-1β, IL-6, and TNF-α) and oxidative stress to prevent extensive tissue damage during inflammation (84, 94–96). IL-10 enhances microvascular supply, tissue oxygenation, and airway epithelium regeneration in allografts through the surface expression of TSG-6, further supporting the therapeutic benefits during wound healing and tissue repair (46, 65, 66, 81). The relationship between inflammation and fibrogenesis has led to IL-10 being identified as a potential antifibrotic target as well as a gatekeeper of fibrotic/antifibrotic signaling, so immune and cell-based therapies aiming to capitalize on IL-10 as a target could be effective in treating lung transplanted patients suffering from delayed would healing.

## Tregs monitoring in clinical lung transplantation

However, numerous clinical studies have been investigating various biomarkers of CLAD to further improve diagnosis, and to characterize early biological processes that lead to the progression of CLAD. Presence of Tregs post lung transplantation have been documented both in peripheral blood and Bronchoalveolar lavage (BAL) samples with varying frequencies, which affected several clinical variables (97, 98). There is little information available about the long-term evolution of peripheral Tregs after lung transplantation. The aim of this review is to discuss the Treg long-term kinetics in lung transplant recipients and their relationship with several clinical variables. As reported, patients with chronic rejection had a significantly lower abundance of peripheral Tregs, while patients without chronic rejection had a significantly higher level of peripheral Tregs (99). Furthermore, these peripheral Tregs were capable of suppressing T cell proliferation and releasing IL-10 *in vitro (* 100). In an investigation of peripheral blood mononuclear cells from lung transplant patients, Bharat and colleagues showed that chronic rejection was associated with a decrease in peripheral Tregs and CD4^+^ T cells producing IL-10. In addition, Tregs were found to induce these IL-10^+^ T cells *in vitro (* 101). In a prospective study, the Hannover group reported the results that Tregs were associated with freedom from chronic lung allograft dysfunction at both early and late time points after transplantation (102). In a study conducted 3 weeks after transplantation, the CD4^+^CD25^high^CD127^Lo^ Treg phenotype was found to inhibit chronic lung allograft dysfunction (102). Moreover, peripheral blood monocyte-derived dendritic cells of lung transplant patients without BOS expressed higher levels of (indolamine oxidase) IDO than those with BOS. It may be of even greater importance to point out that these IDO-expressing dendritic cells were capable of expanding regulatory T cells (103). Clinical studies also investigated the BAL and concluded that Treg abundance varied considerably between individuals with acute rejection and those without, and conflicting associations were identified (97, 104). It has been found, however, that decreased Treg counts in the BAL specimens are associated with BOS in a very small percentage of patients (99).

An analysis of the long-term peripheral kinetics of Tregs was performed by Piloni et al. to determine the association between Tregs and different clinical variables following lung transplantation (98). In previous studies, peripheral Tregs were found to be an important regulatory subset of lung transplant recipients. A recent study confirmed the role of Tregs in lung graft acceptance and rejection. There was a significant decline in peripheral Treg counts in CLAD patients, which demonstrated a significant correlation between the degree of this decrease and the severity of BOS (98). Lung transplants (with BOS) had significantly lower peripheral Tregs than clinically stable lung recipients, and peripheral Tregs early after Lung transplantations are responsible for a protective effect against CLAD, which is associated with a drop in Tregs, T_IL-10_ cells, and an upregulation of T_IFN-γ_ cells in Lung transplant patients (98, 101, 105). In clinical lung transplantation, the assessment of Tregs has emerged first as a tool to predict the progression of CLAD (100, 106–108), FOXP3 activation, and subsequent increase in IL-10 production has been reported in patients with stable lung functions compared to CLAD patients (103). Besides, stable patients also showed increased expression of IDO, which converts tryptophan-kynurenine, and this IDO activity has been therapeutically associated with tolerance in part through direct inhibition of T cell proliferation (109). Conversely, high plasma levels of kynurenine-tryptophan—reflecting high IDO expression—were reported in BOS patients compared to stable patients (110).

Other clinical studies also demonstrated T cell subsets within the lung and reported that patients with acute rejection had lower CD3^+^ cells that expressed FOXP3 compared to non-rejectors (97, 104, 111, 112). At present, most clinical settings still use Donor-specific antibodies (DSA) as an only biomarker in clinical testing. A prospective cohort study of 138 patients performed Tregs analyses from peripheral blood before the transplant procedure and up to the two-year after transplantation. Treg (CD4^+^CD25^high^) data demonstrated that 23% of total recruited patients reflected CLAD symptoms within the two-year after transplantation, but there was no statistical difference was reported between the CLAD-free and CLAD developing patients. However, there was significant increase in the population of CD127^low^, FOXP3^+^, IL-2^+^ and CD152^+^ cells were recorded in the CLAD-free group within three-weeks post-lung transplantation. These findings suggested that increasing levels of CD25^high^CD127^low^, CD25^high^FOXP3^+^, and CD25^high^IL-2^+ of^ CD4^+^ T cell phenotypes with three-weeks after lung transplantation were recognized as a protective mechanism against the progression of CLAD (102).

Another clinical study reported a decrease in peripheral blood Tregs (CD4^+^CD25^high^) in BOS patients compared to patients with stable lung function (100). A subsequent study also demonstrated that drop in Treg (CD4^+^CD25^high^CD127^−^) counts was directly correlated with an increased risk to CLAD progression, and frequency of Treg population was associated with the severity of BOS progression (98). Other clinical studies further demonstrated an increase in Tregs (CD3^+^CD4^+^CD25^high^CD69^−^) in peripheral blood and BAL compared to those in stable lung transplant recipients. This study concluded that stable and evolutive obstructive bronchitis (OB) were dominated by a Treg, Th1, and Th2 activation, however, compared to evolutive OB, Treg and Th2 cells predominated in stable OB conditions, which speculate that Treg could offset the Th activation seen in evolving OB and participate in maintenance of airway obstruction (113).

Similarly, another clinical study also reported the occurrence of low CD4^+^FOXP3^+^ cells in BAL samples collected from who later developed BOS (99). Besides, a higher level of regulatory CCR7^+^CD3^+^CD4^+^CD25^high^FOXP3^+^CD45RA^−^ T cells, which were found protective against the progression of BOS reported in lung transplant recipients (114).

In a recent investigation, CD15s was identified as a specific marker of FOXP3^+^ effector Tregs, which suppress the immune system. An accumulation of CD15s^+^11Tregs was reported in BAL following lung transplantation, and a comparison was made between the numbers of CD15s^+^Tregs in BAL and those in blood. It was demonstrated that long-term lung transplant survivors accumulate a subset of Tregs expressing CD15s in the BAL, but not in the blood (115).

## Tregs mediated immunotherapy

Treg is a potential therapeutic option for the targeted induction and preservation of immunotolerance (116), which is accomplished by the removal of alloreactive T effector cells, or by polarizing alloreactive effector T cells-Tregs ratio in favor of Tregs to suppress alloreactive T effector cells, and subdue graft associated injuries (17, 117, 118). Most of the current immunosuppressive options are inadequate to control early damage to microcirculation resulting in poor long-term outcomes, and therefore both preclinical and clinical data hold great promise to improve long-term outcomes post-transplantation. Several studies have shown the immunosuppressive and therapeutic efficacy of Tregs in various preclinical disease models to treat transplant-related complications (17–19, 66). The direct and indirect therapeutic benefits of Tregs have been investigated in clinical and preclinical studies (17, 18, 119), which echoed that Treg-mediated immunosuppression has been a promising area of cell-based immunotherapy for solid organ transplants (26, 37, 120, 121). Clinical studies adopting Treg mediated immunotherapy in various diseases including type 1 diabetes in children (37), and living donor liver and kidney transplantation have shown that selective augmentation of Tregs can be an effective strategy for promoting transplantation (122).

Transplantation is the last option to rescue end-stage organ failure, which is heavily dependent on the immunosuppressive (IS) medications to protect the graft against alloimmune injury. The IS drugs are non-specific, and therefore cause global immunosuppression and chronic toxicity. It is widely demonstrated that Tregs modulate alloimmune responsiveness and immunosuppress through both TCR-dependent and TCR-independent mechanisms, therefore play a vital role in maintaining immunotolerance. Treg-mediated therapy to be a promising option to taper the magnitude of immunosuppression in transplanted patients for a better long-term graft survival. There is an overwhelming preclinical data in various mouse models of transplantation have demonstrated the efficacy and safety of using Tregs in transplantation settings (11, 38, 123–127). Besides, recent clinical trials using Treg-based therapies in solid organ transplantation also offer the potential of an improved therapeutic efficacy. Although, Tregs are a promising option but the success of Treg based therapy is marred by various limitations. Several cell surface markers have been tested to isolate high purity Tregs from both peripheral/cord blood, selected Tregs should retain their phenotype: of CD4^+^CD127^low^CD25^+^FOXP3^+^CD62L^hi^CCR7^+^ T expressing phenotype for an effective therapeutic candidate. Besides, these phenotype Tregs also display high and sustained FOXP3 and Helios expression and expanded cells should be able to suppress adult peripheral blood T cell proliferation in co-culture assays, retain their purity >95% & viability >90%. In most clinical trials of solid organ transplantation, varying doses of mainly polyclonal Tregs (0.5M-7M) have been tested successfully without any side effects (128). The clinical efficacy and safety of Treg mediated immunotherapy has been successfully tested in liver and kidney transplantation, but not yet in lung transplantation (39, 129). However, e*x vivo* delivery of regulatory T cells for control of alloimmune priming in the donor lung has been tested under pretransplant conditions, which concluded that pre-transplant Treg administration can inhibit alloimmunity within the lung allograft at early time points post-transplant (40).

## Future research

The use of Treg-based immunotherapy to promote tolerance in various solid organ transplantations has emerged as a promising approach. The number, metabolism and function of Treg cells are tightly regulated by numerous costimulatory signals and the associated cytokine signaling (130). As a result, it keeps a delicate balance between immunosuppression and excessive immune activation or autoimmunity. Apart from polyclonal Tregs, there are currently numerous new techniques that have been adopted to make produce antigen specific Tregs *in vitro*, which are therapeutically more effective than polyclonal Tregs. Besides, Chimeric Antigen Receptor (CAR)-expressing Tregs and engineered TCRs, and overexpression of FOXP3 platforms have been introduced to produce antigen-specific Tregs, and preclinical results recorded very encouraging results (131–133). Various clinical trials remain compromised by an inability to manufacture a sufficient Treg cell dose; therefore, it is essential to harness the reparative and regulatory potential of Tregs *in-vivo*. In preclinical studies, a variety of therapeutic options have been used to expand Tregs *in vivo*, including costimulatory and coinhibitory signals, such as abatacept/belatacept primary target CD28, CTLA4, PD-1, ICOS, cytokine signaling, and CAR-Tregs (134–137). Blocking CD28/CTLA4-B7 and CD40-CD154 is one of the most extensively studied costimulation pathways using CTLA4-Ig and MR1 (138–141). CTLA4-Ig, alone or in combination with TCR ligation, exhibits therapeutic efficacy by conversing naive T cells into FOXP3^+^ T cells and by expanding their numbers, thus favoring graft survival (142). Several preclinical studies led to the development of abatacept, which has now been approved for the prevention of Graft-versus-host disease GvHD (143). IL-2 plays a vital role in Treg generation, survival, stability, and function, and signaling via the IL-2 receptor and activation of STAT5 signaling pathways can be utilized to promote Treg expansion *in vivo (* 144, 145). A number of mediators have been reported to stimulate Tregs, which include TSG-6, TGF-β, IL-5, IL-9, IL-10, IL-27, IL-35 and IL-33 to facilities immune tolerance and repair process (54, 146–151). In addition to cytokines, some growth factors, especially TSG-6, play crucial role in modulating Treg levels during tissue repair. TSG-6 has been tested extensively in preclinical studies, which demonstrated its positive effects on wound healing and tissue repair (81, 84, 91, 152). Besides, the currently available preclinical data indicates that TSG-6 can function as a potential antifibrotic and angiogenic agent and can regulate pro-inflammatory cytokines and enhance tissue repair in multiple animal models, while suppressing inflammatory reactions induced by ischemia in a variety of disease models (54, 65, 66, 91, 153). In addition to costimulatory and cytokine signaling, CAR-Tregs have been investigated to generate antigen-specific Tregs by expanding Tregs with APCs and specific antigens or engineering them with T-cell receptors. TCR-engineered Tregs are promising, but they are still MHC-restricted, limiting individual patient application (132, 133). The single-chain variable fragment, extracellular hinge, transmembrane region, and intracellular signaling domains are used in an MHC-independent way to engineer Tregs with chimeric antigen receptor genes. In animal models, CAR-Tregs have shown great potential for treating different diseases, especially allograft rejection and various autoimmune diseases (154, 155), and CAR-Tregs are a potential choice of immunetolerance in clinical transplantation to achieve an effective immunosuppression (154).

## Clinical limitations

Several preclinical studies have suggested that Treg infusion after lung transplantation may reduce acute and chronic rejection. In humans, Treg therapy may be substantially limited by several potential pitfalls. A crucial question remains, however, as to whether Treg infusions following lung transplants are safe for humans. Several early phase trials discussed above suggest that there will be no adverse effects associated with Treg infusion after lung transplantation, although no clinical trials have been initiated. Despite these vital regulatory and reparative effects of Tregs in preclinical and clinical transplantation, Treg therapy still faces crucial challenges, which include how to calculate an effective dose, antigen specificity, expansion, and large-scale production for future clinical trials.

## Conclusion

We conclude that Tregs are a vital part of the immune response and play a major role in determining the transplant functioning in clinical transplantations, and FOXP3^+^ Tregs may serve as a relevant biomarker for predicting outcomes of transplantation.

## Author contributions

All authors listed have made a substantial, direct, and intellectual contribution to the work, and approved it for publication.

## References

[1] Luckraz H Goddard M McNeil K Atkinson C Charman SC Stewart S. Microvascular changes in small airways predispose to obliterative bronchiolitis after lung transplantation. J Heart Lung Transplant (2004) 23(5):527–31. doi: 10.1016/j.healun.2003.07.003 15135366

[2] Wigfield CH Buie V Onsager D. “Age” in lung transplantation: factors related to outcomes and other considerations. Curr Pulmonology Rep (2016) 5:152–8. doi: 10.1007/s13665-016-0151-y PMC4992499 27610336

[3] Boucek MM Waltz DA Edwards LB Taylor DO Keck BM Trulock EP. Registry of the international society for heart and lung transplantation: ninth official pediatric heart transplantation report–2006. J Heart Lung Transplant (2006) 25(8):893–903. doi: 10.1016/j.healun.2006.05.014 16890109

[4] Bos S Vos R Van Raemdonck DE Verleden GM. Survival in adult lung transplantation: where are we in 2020? Curr Opin Organ Transplant (2020) 25(3):268–73. doi: 10.1097/mot.0000000000000753 32332197

[5] Gauthier JM Li W Hsiao HM Takahashi T Arefanian S Krupnick AS. Mechanisms of graft rejection and immune regulation after lung transplant. Ann Am Thorac Soc (2017) 14(Supplement_3):S216–S9. doi: 10.1513/AnnalsATS.201607-576MG PMC5711341 28945475

[6] Chiu S Fernandez R Subramanian V Sun H DeCamp MM Kreisel D. Lung injury combined with loss of regulatory T cells leads to *de novo* lung-restricted autoimmunity. J Immunol (2016) 197(1):51–7. doi: 10.4049/jimmunol.1502539 PMC4912911 27194786

[7] Khan MA Jiang X Dhillon G Beilke J Holers VM Atkinson C. Cd4+ T cells and complement independently mediate graft ischemia in the rejection of mouse orthotopic tracheal transplants. Circ Res (2011) 109(11):1290–301. doi: 10.1161/CIRCRESAHA.111.250167 PMC3243047 21998328

[8] Hsu JL Jiang X Khan MA Sobel RA Clemons KV Stevens DA. Aspergillus invasion increases with progressive airway ischemia. Ann Am Thorac Soc (2014) 11(Supplement 1):S79–S. doi: 10.1513/AnnalsATS.201307-240MG PMC3796538 24155924

[9] Khan MA Nicolls MR. Complement-mediated microvascular injury leads to chronic rejection. Adv Exp Med Biol (2013) 734:233–46. doi: 10.1007/978-1-4614-4118-2_16 PMC4015512 23402031

[10] Ansari AW Khan MA Schmidt RE Broering DC. Harnessing the immunotherapeutic potential of T-lymphocyte co-signaling molecules in transplantation. Immunol Lett (2017) 183:8–16. doi: 10.1016/j.imlet.2017.01.008 28119073

[11] Khan MA. Regulatory T cells mediated immunomodulation during asthma: A therapeutic standpoint. J Trans Med (2020) 18(1):456. doi: 10.1186/s12967-020-02632-1 PMC7713035 33267824

[12] Khan MA Hsu JL Assiri AM Broering DC. Targeted complement inhibition and microvasculature in transplants: A therapeutic perspective. Clin Exp Immunol (2015) 183:175–86. doi: 10.1111/cei.12713 PMC4711168 26404106

[13] Sakaguchi S Powrie F. Emerging challenges in regulatory T cell function and biology. Science (2007) 317(5838):627–9. doi: 10.1126/science.1142331 17673654

[14] Khan MA Moeez S Akhtar S. T-regulatory cell-mediated immune tolerance as a potential immunotherapeutic strategy to facilitate graft survival. Blood Transfus (2013) 11(3):357–63. doi: 10.2450/2013.0258-12 PMC3729125 23736920

[15] Khan MA. T regulatory cell mediated immunotherapy for solid organ transplantation: A clinical perspective. Mol Med (2016) 22:892–904. doi: 10.2119/molmed.2016.00050 PMC5319206 27878210

[16] Khan MA Shamma T. Complement factor and T-cell interactions during alloimmune inflammation in transplantation. J Leukoc Biol (2019) 105(4):681–94. doi: 10.1002/JLB.5RU0718-288R 30536904

[17] Khan MA Alanazi F Ahmed HA Al-Mohanna FH Assiri AM Broering DC. Foxp3+ Regulatory T cell ameliorates microvasculature in the rejection of mouse orthotopic tracheal transplants. Clin Immunol (2017) 174:84–98. doi: 10.1016/j.clim.2016.11.011 27939405

[18] Khan MA Alanazi F Ahmed HA Shamma T Kelly K Hammad MA. Ipsc-derived msc therapy induces immune tolerance and supports long-term graft survival in mouse orthotopic tracheal transplants. Stem Cell Res Ther (2019) 10(1):290. doi: 10.1186/s13287-019-1397-4 31547869 PMC6757436

[19] Khan MA Alanazi F Ahmed HA Vater A Assiri AM Broering DC. C5a blockade increases regulatory T cell numbers and protects against microvascular loss and epithelial damage in mouse airway allografts. Front Immunol (2018) 9:1010(1010). doi: 10.3389/fimmu.2018.01010 29881374 PMC5976734

[20] Khan MA Maasch C Vater A Klussmann S Morser J Leung LL. Targeting complement component 5a promotes vascular integrity and limits airway remodeling. Proc Natl Acad Sci U.S.A. (2013) 110(15):6061–6. doi: 10.1073/pnas.12179911101217991110 PMC3625314 23530212

[21] Bilate AM Lafaille JJ. Induced cd4+Foxp3+ Regulatory T cells in immune tolerance. Annu Rev Immunol (2012) 30:733–58. doi: 10.1146/annurev-immunol-020711-075043 22224762

[22] Josefowicz SZ Lu LF Rudensky AY. Regulatory T cells: mechanisms of differentiation and function. Annu Rev Immunol (2012) 30:531–64. doi: 10.1146/annurev.immunol.25.022106.141623 PMC6066374 22224781

[23] Sakaguchi S. Naturally arising foxp3-expressing cd25+Cd4+ Regulatory T cells in immunological tolerance to self and non-self. Nat Immunol (2005) 6(4):345–52. doi: 10.1038/ni1178 15785760

[24] Arroyo Hornero R Betts GJ Sawitzki B Vogt K Harden PN Wood KJ. Cd45ra distinguishes cd4+Cd25+Cd127-/low tsdr demethylated regulatory T cell subpopulations with differential stability and susceptibility to tacrolimus-mediated inhibition of suppression. Transplantation (2017) 101(2):302–9. doi: 10.1097/TP.0000000000001278 PMC5265687 28118317

[25] Santegoets SJ Dijkgraaf EM Battaglia A Beckhove P Britten CM Gallimore A. Monitoring regulatory T cells in clinical samples: consensus on an essential marker set and gating strategy for regulatory T cell analysis by flow cytometry. Cancer Immunol Immunother (2015) 64(10):1271–86. doi: 10.1007/s00262-015-1729-x PMC4554737 26122357

[26] Bluestone JA. Foxp3, the transcription factor at the heart of the rebirth of immune tolerance. J Immunol (2017) 198(3):979–80. doi: 10.4049/jimmunol.1602060 28115585

[27] Trojan K Unterrainer C Weimer R Bulut N Morath C Aly M. Helios expression and foxp3 tsdr methylation of ifny+ and ifny- treg from kidney transplant recipients with good long-term graft function. PloS One (2017) 12(3):e0173773. doi: 10.1371/journal.pone.0173773 28296931 PMC5351987

[28] Alvarez Salazar EK Cortes-Hernandez A Aleman-Muench GR Alberu J Rodriguez-Aguilera JR Recillas-Targa F. Methylation of foxp3 tsdr underlies the impaired suppressive function of tregs from long-term belatacept-treated kidney transplant patients. Front Immunol (2017) 8:219. doi: 10.3389/fimmu.2017.00219 28316600 PMC5334349

[29] Colamatteo A Carbone F Bruzzaniti S Galgani M Fusco C Maniscalco GT. Molecular mechanisms controlling foxp3 expression in health and autoimmunity: from epigenetic to post-translational regulation. Front Immunol (2019) 10:3136. doi: 10.3389/fimmu.2019.03136 32117202 PMC7008726

[30] Freudenberg K Lindner N Dohnke S Garbe AI Schallenberg S Kretschmer K. Critical role of tgf-beta and il-2 receptor signaling in foxp3 induction by an inhibitor of DNA methylation. Front Immunol (2018) 9:125. doi: 10.3389/fimmu.2018.00125 29456534 PMC5801288

[31] Miyara M Yoshioka Y Kitoh A Shima T Wing K Niwa A. Functional delineation and differentiation dynamics of human cd4+ T cells expressing the foxp3 transcription factor. Immunity (2009) 30(6):899–911. doi: 10.1016/j.immuni.2009.03.019 19464196

[32] Deng G Song X Fujimoto S Piccirillo CA Nagai Y Greene MI. Foxp3 post-translational modifications and treg suppressive activity. Front Immunol (2019) 10:2486. doi: 10.3389/fimmu.2019.02486 31681337 PMC6813729

[33] Nair VS Song MH Ko M Oh KI. DNA demethylation of the foxp3 enhancer is maintained through modulation of ten-eleven-translocation and DNA methyltransferases. Mol Cells (2016) 39(12):888–97. doi: 10.14348/molcells.2016.0276 PMC5223106 27989104

[34] Bhorade SM Stern E. Immunosuppression for lung transplantation. Proc Am Thorac Soc (2009) 6(1):47–53. doi: 10.1513/pats.200808-096GO 19131530

[35] McDermott JK Girgis RE. Individualizing immunosuppression in lung transplantation. Glob Cardiol Sci Pract (2018) 2018(1):5. doi: 10.21542/gcsp.2018.5 29644232 PMC5857067

[36] Furukawa A Wisel SA Tang Q. Impact of immune-modulatory drugs on regulatory T cell. Transplantation (2016) 100(11):2288–300. doi: 10.1097/tp.0000000000001379 PMC5077666 27490409

[37] Marek-Trzonkowska N Mysliwiec M Dobyszuk A Grabowska M Techmanska I Juscinska J. Administration of cd4+Cd25highcd127- regulatory T cells preserves beta-cell function in type 1 diabetes in children. Diabetes Care (2012) 35(9):1817–20. doi: 10.2337/dc12-0038 PMC3425004 22723342

[38] Jiang S Tsang J Lechler RI. Adoptive cell therapy using in vitro generated human cd4+ Cd25+ Regulatory T cells with indirect allospecificity to promote donor-specific transplantation tolerance. Transplant Proc (2006) 38(10):3199–201. doi: 10.1016/j.transproceed.2006.10.132 17175221

[39] Sanchez-Fueyo A Whitehouse G Grageda N Cramp ME Lim TY Romano M. Applicability, safety, and biological activity of regulatory T cell therapy in liver transplantation. Am J Transplant (2020) 20(4):1125–36. doi: 10.1111/ajt.15700 PMC7154724 31715056

[40] Miyamoto E Takahagi A Ohsumi A Martinu T Hwang D Boonstra KM. Ex vivo delivery of regulatory T cells for control of alloimmune priming in the donor lung. Eur Respir J (2021) 59(1–13). doi: 10.1183/13993003.00798-2021 34475226

[41] van Kooten C. Counteracting dysfunction of regulatory T cells in organ transplantation. Proc Natl Acad Sci U.S.A. (2017) 114(27):6883–4. doi: 10.1073/pnas.1708493114 PMC5502658 28642347

[42] Camirand G Riella LV. Treg-centric view of immunosuppressive drugs in transplantation: A balancing act. Am J Transplant (2017) 17(3):601–10. doi: 10.1111/ajt.14029 27581661

[43] Werner S Grose R. Regulation of wound healing by growth factors and cytokines. Physiol Rev (2003) 83(3):835–70. doi: 10.1152/physrev.2003.83.3.835 12843410

[44] Brancato SK Albina JE. Wound macrophages as key regulators of repair: origin, phenotype, and function. Am J Pathol (2011) 178(1):19–25. doi: 10.1016/j.ajpath.2010.08.003 21224038 PMC3069845

[45] Chung AS Ferrara N. Developmental and pathological angiogenesis. Annu Rev Cell Dev Biol (2011) 27:563–84. doi: 10.1146/annurev-cellbio-092910-154002 21756109

[46] King A Balaji S Le LD Crombleholme TM Keswani SG. Regenerative wound healing: the role of interleukin-10. Adv Wound Care (2014) 3(4):315–23. doi: 10.1089/wound.2013.0461 PMC3985521 24757588

[47] Stellos K Kopf S Paul A Marquardt JU Gawaz M Huard J. Platelets in regeneration. Semin Thromb Hemost (2010) 36(2):175–84. doi: 10.1055/s-0030-1251502 20414833

[48] Nosbaum A Prevel N Truong HA Mehta P Ettinger M Scharschmidt TC. Cutting edge: regulatory T cells facilitate cutaneous wound healing. J Immunol (2016) 196(5):2010–4. doi: 10.4049/jimmunol.1502139 PMC4761457 26826250

[49] Wilgus TA. Immune cells in the healing skin wound: influential players at each stage of repair. Pharmacol Res (2008) 58(2):112–6. doi: 10.1016/j.phrs.2008.07.009 18723091

[50] Larouche J Sheoran S Maruyama K Martino MM. Immune regulation of skin wound healing: mechanisms and novel therapeutic targets. Adv Wound Care (New Rochelle) (2018) 7(7):209–31. doi: 10.1089/wound.2017.0761 PMC6032665 29984112

[51] MacLeod AS Mansbridge JN. The innate immune system in acute and chronic wounds. Adv Wound Care (New Rochelle) (2016) 5(2):65–78. doi: 10.1089/wound.2014.0608 26862464 PMC4742992

[52] Tosello-Trampont A Surette FA Ewald SE Hahn YS. Immunoregulatory role of nk cells in tissue inflammation and regeneration. Front Immunol (2017) 8:301. doi: 10.3389/fimmu.2017.00301 28373874 PMC5357635

[53] Chen L Deng H Cui H Fang J Zuo Z Deng J. Inflammatory responses and inflammation-associated diseases in organs. Oncotarget (2018) 9(6):7204–18. doi: 10.18632/oncotarget.23208 PMC5805548 29467962

[54] Kota DJ Wiggins LL Yoon N Lee RH. Tsg-6 produced by hmscs delays the onset of autoimmune diabetes by suppressing th1 development and enhancing tolerogenicity. Diabetes (2013) 62(6):2048–58. doi: 10.2337/db12-0931 PMC3661629 23349496

[55] Lee RH Pulin AA Seo MJ Kota DJ Ylostalo J Larson BL. Intravenous hmscs improve myocardial infarction in mice because cells embolized in lung are activated to secrete the anti-inflammatory protein tsg-6. Cell Stem Cell (2009) 5(1):54–63. doi: 10.1016/j.stem.2009.05.003 19570514 PMC4154377

[56] Strbo N Yin N Stojadinovic O. Innate and adaptive immune responses in wound epithelialization. Adv Wound Care (New Rochelle) (2014) 3(7):492–501. doi: 10.1089/wound.2012.0435 25032069 PMC4086194

[57] Ucuzian AA Gassman AA East AT Greisler HP. Molecular mediators of angiogenesis. J Burn Care Res (2010) 31(1):158–75. doi: 10.1097/BCR.0b013e3181c7ed82 PMC2818794 20061852

[58] Borges PA Waclawiak I Georgii JL Fraga-Junior VDS Barros JF Lemos FS. Adenosine diphosphate improves wound healing in diabetic mice through P2y12 receptor activation. Front Immunol (2021) 12:651740. doi: 10.3389/fimmu.2021.651740 33828561 PMC8019717

[59] Perez-Aso M Chiriboga L Cronstein BN. Pharmacological blockade of adenosine A2a receptors diminishes scarring. FASEB J (2012) 26(10):4254–63. doi: 10.1096/fj.12-209627 PMC3448776 22767233

[60] Khan MA Shamma T Kazmi S Altuhami A Ahmed HA Assiri AM. Hypoxia-induced complement dysregulation is associated with microvascular impairments in mouse tracheal transplants. J Transl Med (2020) 18(1):147. doi: 10.1186/s12967-020-02305-z 32234039 PMC7110829

[61] Sakai R Ito M Komai K Iizuka-Koga M Matsuo K Nakayama T. Kidney gata3(+) regulatory T cells play roles in the convalescence stage after antibody-mediated renal injury. Cell Mol Immunol (2021) 18(5):1249–61. doi: 10.1038/s41423-020-00547-x PMC8093306 32917984

[62] Faustino LD Griffith JW Rahimi RA Nepal K Hamilos DL Cho JL. Interleukin-33 activates regulatory T cells to suppress innate gammadelta T cell responses in the lung. Nat Immunol (2020) 21(11):1371–83. doi: 10.1038/s41590-020-0785-3 PMC7578082 32989331

[63] Carney K Chang YR Wilson S Calnan C Reddy PS Chan WY. Regulatory T-cell-intrinsic amphiregulin is dispensable for suppressive function. J Allergy Clin Immunol (2016) 137(6):1907–9. doi: 10.1016/j.jaci.2016.01.030 PMC4889774 27040371

[64] Murai M Turovskaya O Kim G Madan R Karp CL Cheroutre H. Interleukin 10 acts on regulatory T cells to maintain expression of the transcription factor foxp3 and suppressive function in mice with colitis. Nat Immunol (2009) 10(11):1178–84. doi: 10.1038/ni.1791 PMC2898179 19783988

[65] Kazmi S Khan MA Shamma T Altuhami A Ahmed HA Mohammed Assiri A. Targeting interleukin-10 restores graft microvascular supply and airway epithelium in rejecting allografts. Int J Mol Sci (2022) 23(3):1269. doi: 10.3390/ijms23031269 35163192 PMC8836023

[66] Khan MA Ashoor GA Shamma T Alanazi F Altuhami A Kazmi S. Il-10 mediated immunomodulation limits subepithelial fibrosis and repairs airway epithelium in rejecting airway allografts. Cells (2021) 10(5):1248. doi: 10.3390/cells10051248 34069395 PMC8158696

[67] Hsu P Santner-Nanan B Hu M Skarratt K Lee CH Stormon M. Il-10 potentiates differentiation of human induced regulatory T cells via stat3 and foxo1. J Immunol (2015) 195(8):3665–74. doi: 10.4049/jimmunol.1402898 26363058

[68] Lopes RL Borges TJ Zanin RF Bonorino C. Il-10 is required for polarization of macrophages to M2-like phenotype by mycobacterial dnak (Heat shock protein 70). Cytokine (2016) 85:123–9. doi: 10.1016/j.cyto.2016.06.018 27337694

[69] Makita N Hizukuri Y Yamashiro K Murakawa M Hayashi Y. Il-10 enhances the phenotype of M2 macrophages induced by il-4 and confers the ability to increase eosinophil migration. Int Immunol (2015) 27(3):131–41. doi: 10.1093/intimm/dxu090 25267883

[70] Cypel M Liu M Rubacha M Yeung JC Hirayama S Anraku M. Functional repair of human donor lungs by il-10 gene therapy. Sci Trans Med (2009) 1(4):4ra9–9. doi: 10.1126/scitranslmed.3000266 20368171

[71] Steen EH Wang X Balaji S Butte MJ Bollyky PL Keswani SG. The role of the anti-inflammatory cytokine interleukin-10 in tissue fibrosis. Adv Wound Care (New Rochelle) (2020) 9(4):184–98. doi: 10.1089/wound.2019.1032 PMC7047112 32117582

[72] Boehler A. The role of interleukin-10 in lung transplantation. Transpl Immunol (2002) 9(2-4):121–4. doi: 10.1016/S0966-3274(02)00045-X 12180818

[73] Deng B Wehling-Henricks M Villalta SA Wang Y Tidball JG. Il-10 triggers changes in macrophage phenotype that promote muscle growth and regeneration. J Immunol (2012) 189(7):3669–80. doi: 10.4049/jimmunol.1103180 PMC3448810 22933625

[74] Hara M Kingsley CI Niimi M Read S Turvey SE Bushell AR. Il-10 is required for regulatory T cells to mediate tolerance to alloantigens in vivo. J Immunol (2001) 166(6):3789–96. doi: 10.4049/jimmunol.166.6.3789 11238621

[75] Hawrylowicz CM. Regulatory T cells and il-10 in allergic inflammation. J Exp Med (2005) 202(11):1459–63. doi: 10.1084/jem.20052211 PMC2213335 16330811

[76] Tang-Feldman YJ Lochhead GR Lochhead SR Yu C Pomeroy C. Interleukin-10 repletion suppresses pro-inflammatory cytokines and decreases liver pathology without altering viral replication in murine cytomegalovirus (Mcmv)-infected il-10 knockout mice. Inflammation Res (2011) 60(3):233–43. doi: 10.1007/s00011-010-0259-4 PMC3036806 20922456

[77] Niu J Yue W Song Y Zhang Y Qi X Wang Z. Prevention of acute liver allograft rejection by il-10-engineered mesenchymal stem cells. Clin Exp Immunol (2014) 176(3):473–84. doi: 10.1111/cei.12283 PMC4008992 24527865

[78] Fischbein MP Yun J Laks H Irie Y Oslund-Pinderski L Fishbein MC. Regulated interleukin-10 expression prevents chronic rejection of transplanted hearts. J Thorac Cardiovasc Surg (2003) 126(1):216–23. doi: 10.1016/S0022-5223(03)00026-6 12878958

[79] Rachmawati H Beljaars L Reker-Smit C Bakker HI Van Loenen-Weemaes AM Lub-De Hooge MN. Intravenous administration of recombinant human il-10 suppresses the development of anti-thy 1-induced glomerulosclerosis in rats. PDA J Pharm Sci Technol (2011) 65(2):116–30. 21502073

[80] O’Garra A Vieira PL Vieira P Goldfeld AE. Il-10–producing and naturally occurring cd4+ Tregs: limiting collateral damage. J Clin Invest (2004) 114(10):1372–8. doi: 10.1172/JCI23215 PMC525746 15545984

[81] Shakya S Mack JA Alipour M Maytin EV. Cutaneous wounds in mice lacking tsg-6 exhibit delayed closure and an abnormal inflammatory response. J Invest Dermatol (2020) 140(12):2505–14. doi: 10.1016/j.jid.2020.04.015 PMC7749718 32422216

[82] Heim C Khan MA von Silva-Tarouca B Kuckhahn A Stamminger T Ramsperger-Gleixner M. Preservation of microvascular integrity in murine orthotopic tracheal allografts by clopidogrel. Transplantation (2019) 103(899–908). doi: 10.1097/tp.0000000000002571 30801550

[83] Sharir R Semo J Shaish A Landa-Rouben N Entin-Meer M Keren G. Regulatory T cells influence blood flow recovery in experimental hindlimb ischaemia in an il-10-dependent manner. Cardiovasc Res (2014) 103(4):585–96. doi: 10.1093/cvr/cvu159 24966183

[84] Liu L Song H Duan H Chai J Yang J Li X. Tsg-6 secreted by human umbilical cord-mscs attenuates severe burn-induced excessive inflammation via inhibiting activations of P38 and jnk signaling. Sci Rep (2016) 6:30121. doi: 10.1038/srep30121 27444207 PMC4957124

[85] Mittal M Tiruppathi C Nepal S Zhao Y-Y Grzych D Soni D. Tnfα-stimulated gene-6 (Tsg6) activates macrophage phenotype transition to prevent inflammatory lung injury. Proc Natl Acad Sci (2016) 113(50):E8151–E8. doi: 10.1073/pnas.1614935113 PMC5167170 27911817

[86] Choi H Lee RH Bazhanov N Oh JY Prockop DJ. Anti-inflammatory protein tsg-6 secreted by activated mscs attenuates zymosan-induced mouse peritonitis by decreasing tlr2/nf-kappab signaling in resident macrophages. Blood (2011) 118(2):330–8. doi: 10.1182/blood-2010-12-327353 PMC3138686 21551236

[87] Dyer DP Salanga CL Johns SC Valdambrini E Fuster MM Milner CM. The anti-inflammatory protein tsg-6 regulates chemokine function by inhibiting chemokine/glycosaminoglycan interactions. J Biol Chem (2016) 291(24):12627–40. doi: 10.1074/jbc.M116.720953 PMC4933465 27044744

[88] Getting SJ Mahoney DJ Cao T Rugg MS Fries E Milner CM. The link module from human tsg-6 inhibits neutrophil migration in a hyaluronan- and inter-alpha -inhibitor-independent manner. J Biol Chem (2002) 277(52):51068–76. doi: 10.1074/jbc.M205121200 12401803

[89] Watanabe R Sato Y Ozawa N Takahashi Y Koba S Watanabe T. Emerging roles of tumor necrosis factor-stimulated gene-6 in the pathophysiology and treatment of atherosclerosis. Int J Mol Sci (2018) 19(2):465. doi: 10.3390/ijms19020465 29401724 PMC5855687

[90] Hu Y Li G Zhang Y Liu N Zhang P Pan C. Upregulated tsg-6 expression in adscs inhibits the bv2 microglia-mediated inflammatory response. BioMed Res Int (2018) 2018:7239181. doi: 10.1155/2018/7239181 30584538 PMC6280241

[91] Kui L Chan GC Lee PP. Tsg-6 downregulates ifn-alpha and tnf-alpha expression by suppressing irf7 phosphorylation in human plasmacytoid dendritic cells. Mediators Inflammation (2017) 2017:7462945. doi: 10.1155/2017/7462945 PMC5358455 28367002

[92] Milner CM Day AJ. Tsg-6: A multifunctional protein associated with inflammation. J Cell Sci (2003) 116(Pt 10):1863–73. doi: 10.1242/jcs.00407 12692188

[93] Yang H Wu L Deng H Chen Y Zhou H Liu M. Anti-inflammatory protein tsg-6 secreted by bone marrow mesenchymal stem cells attenuates neuropathic pain by inhibiting the tlr2/myd88/nf-kappab signaling pathway in spinal microglia. J Neuroinflamm (2020) 17(1):154. doi: 10.1186/s12974-020-1731-x PMC7216552 32393298

[94] Wan YM Wu HM Li YH Xu ZY Yang JH Liu C. Tsg-6 inhibits oxidative stress and induces M2 polarization of hepatic macrophages in mice with alcoholic hepatitis via suppression of stat3 activation. Front Pharmacol (2020) 11:10. doi: 10.3389/fphar.2020.00010 32116692 PMC7010862

[95] Qi Y Jiang D Sindrilaru A Stegemann A Schatz S Treiber N. Tsg-6 released from intradermally injected mesenchymal stem cells accelerates wound healing and reduces tissue fibrosis in murine full-thickness skin wounds. J Invest Dermatol (2014) 134(2):526–37. doi: 10.1038/jid.2013.328 23921952

[96] Guo P Zhang SZ He H Zhu YT Tseng SC. Tsg-6 controls transcription and activation of matrix metalloproteinase 1 in conjunctivochalasis. Invest Ophthalmol Vis Sci (2012) 53(3):1372–80. doi: 10.1167/iovs.11-8738 PMC3339910 22297496

[97] Neujahr DC Cardona AC Ulukpo O Rigby M Pelaez A Ramirez A. Dynamics of human regulatory T cells in lung lavages of lung transplant recipients. Transplantation (2009) 88(4):521–7. doi: 10.1097/TP.0b013e3181b0e719 PMC2773802 19696635

[98] Piloni D Morosini M Magni S Balderacchi A Scudeller L Cova E. Analysis of long term cd4+Cd25highcd127- T-reg cells kinetics in peripheral blood of lung transplant recipients. BMC Pulm Med (2017) 17(1):102. doi: 10.1186/s12890-017-0446-y 28720146 PMC5516333

[99] Bhorade SM Chen H Molinero L Liao C Garrity ER Vigneswaran WT. Decreased percentage of cd4+Foxp3+ Cells in bronchoalveolar lavage from lung transplant recipients correlates with development of bronchiolitis obliterans syndrome. Transplantation (2010) 90(5):540–6. doi: 10.1097/TP.0b013e3181e8dabe PMC4737704 20628341

[100] Meloni F Vitulo P Bianco AM Paschetto E Morosini M Cascina A. Regulatory cd4+Cd25+ T cells in the peripheral blood of lung transplant recipients: correlation with transplant outcome. Transplantation (2004) 77(5):762–6. doi: 10.1097/01.tp.0000116565.86752.6b 15021844

[101] Bharat A Fields RC Steward N Trulock EP Patterson GA Mohanakumar T. Cd4+25+ Regulatory T cells limit th1-autoimmunity by inducing il-10 producing T cells following human lung transplantation. Am J Transplant (2006) 6(8):1799–808. doi: 10.1111/j.1600-6143.2006.01383.x 16889540

[102] Salman J Ius F Knoefel AK Sommer W Siemeni T Kuehn C. Association of Higher Cd4(+) Cd25(High) Cd127(Low) , Foxp3(+) , and Il-2(+) T Cell Frequencies Early after Lung Transplantation with Less Chronic Lung Allograft Dysfunction at Two Years. Am J Transplant (2017) 17(6):1637–48. doi: 10.1111/ajt.14148 27931084

[103] Botturi K Lacoeuille Y Thomas P Boniface S Reynaud-Gaubert M Magnan A. Ctla-4-mediated regulatory phenotype of T-cells in tolerant lung recipients. Eur Respir J (2008) 31(6):1167–76. doi: 10.1183/09031936.00093207 18256061

[104] Gregson AL Hoji A Saggar R Ross DJ Kubak BM Jamieson BD. Bronchoalveolar immunologic profile of acute human lung transplant allograft rejection. Transplantation (2008) 85(7):1056–9. doi: 10.1097/TP.0b013e318169bd85 PMC2744369 18408589

[105] Neujahr DC Larsen CP. Regulatory T cells in lung transplantation–an emerging concept. Semin Immunopathol (2011) 33(2):117–27. doi: 10.1007/s00281-011-0253-0 PMC3395059 21424593

[106] Studer SM George MP Zhu X Song Y Valentine VG Stoner MW. Cd28 down-regulation on cd4 T cells is a marker for graft dysfunction in lung transplant recipients. Am J Respir Crit Care Med (2008) 178(7):765–73. doi: 10.1164/rccm.200701-013OC PMC2556458 18617642

[107] Tai X Cowan M Feigenbaum L Singer A. Cd28 costimulation of developing thymocytes induces foxp3 expression and regulatory T cell differentiation independently of interleukin 2. Nat Immunol (2005) 6(2):152–62. doi: 10.1038/ni1160 15640801

[108] Zeiser R Nguyen VH Beilhack A Buess M Schulz S Baker J. Inhibition of cd4+Cd25+ Regulatory T-cell function by calcineurin-dependent interleukin-2 production. Blood (2006) 108(1):390–9. doi: 10.1182/blood-2006-01-0329 PMC1895845 16522809

[109] Hwu P Du MX Lapointe R Do M Taylor MW Young HA. Indoleamine 2,3-dioxygenase production by human dendritic cells results in the inhibition of T cell proliferation. J Immunol (2000) 164(7):3596–9. doi: 10.4049/jimmunol.164.7.3596 10725715

[110] Meloni F Giuliano S Solari N Draghi P Miserere S Bardoni AM. Indoleamine 2,3-dioxygenase in lung allograft tolerance. J Heart Lung Transplant (2009) 28(11):1185–92. doi: 10.1016/j.healun.2009.07.023 19783182

[111] Gavin MA Torgerson TR Houston E DeRoos P Ho WY Stray-Pedersen A. Single-cell analysis of normal and foxp3-mutant human T cells: foxp3 expression without regulatory T cell development. Proc Natl Acad Sci U.S.A. (2006) 103(17):6659–64. doi: 10.1073/pnas.0509484103 PMC1458937 16617117

[112] Wang J Ioan-Facsinay A van der Voort EI Huizinga TW Toes RE. Transient expression of foxp3 in human activated nonregulatory cd4+ T cells. Eur J Immunol (2007) 37(1):129–38. doi: 10.1002/eji.200636435 17154262

[113] Mamessier E Lorec AM Thomas P Badier M Magnan A Reynaud-Gaubert M. T regulatory cells in stable posttransplant bronchiolitis obliterans syndrome. Transplantation (2007) 84(7):908–16. doi: 10.1097/01.tp.0000281408.20686.cb 17984845

[114] Budding K van de Graaf EA Paantjens AW Kardol-Hoefnagel T Kwakkel-van Erp JM van Kessel DA. Profiling of peripheral blood mononuclear cells does not accurately predict the bronchiolitis obliterans syndrome after lung transplantation. Transpl Immunol (2015) 32(3):195–200. doi: 10.1016/j.trim.2015.03.003 25841614

[115] O'Sullivan BJ Hopkins P Trotter M Fiene A Tan M Sinclair K. Accumulation of intragraft cd15s+Tregs in long-term lung transplant survivors. J Heart Lung Transplant (2018) 37(4, Supplement):S209. doi: 10.1016/j.healun.2018.01.510

[116] Wood KJ Sakaguchi S. Regulatory T cells in transplantation tolerance. Nat Rev Immunol (2003) 3(3):199–210. doi: 10.1038/nri1027nri1027[pii 12658268

[117] Campbell DJ Koch MA. Treg cells: patrolling a dangerous neighborhood. Nat Med (2011) 17(8):929–30. doi: 10.1038/nm.2433 21818088

[118] Tang Q Vincenti F. Transplant trials with tregs: perils and promises. J Clin Invest (2017) 127(7):2505–12. doi: 10.1172/JCI90598 PMC5490750 28665300

[119] Mohammad Afzal Khan FA Ahmed HA Hasan AF Altuhami A Assiri AM Clemens D. The therapeutic potential of treg cells in preserving microvascular health in a mouse model of orthotopic tracheal transplantation. J Clin Cell Immunol (2016) 7(3):89. doi: 10.4172/2155-9899.C1.028

[120] Abdel-Gadir A Massoud AH Chatila TA. Antigen-specific treg cells in immunological tolerance: implications for allergic diseases. F1000Research (2018) 7:38–. doi: 10.12688/f1000research.12650.1 PMC5765398 29375821

[121] Lu L Barbi J Pan F. The regulation of immune tolerance by foxp3. Nat Rev Immunol (2017) 17(11):703–17. doi: 10.1038/nri.2017.75 PMC5793224 28757603

[122] Todo S Yamashita K Goto R Zaitsu M Nagatsu A Oura T. A pilot study of operational tolerance with a regulatory T cell-based cell therapy in living donor liver transplantation. Hepatology (2016) 64(632–643). doi: 10.1002/hep.28459 26773713

[123] Li G Boucher JC Kotani H Park K Zhang Y Shrestha B. 4-1bb enhancement of car T function requires nf-kappab and trafs. JCI Insight (2018) 3(18):1–18. doi: 10.1172/jci.insight.121322 PMC6237232 30232281

[124] Carson WF Guernsey LA Singh A Vella AT Schramm CM Thrall RS. Accumulation of regulatory T cells in local draining lymph nodes of the lung correlates with spontaneous resolution of chronic asthma in a murine model. Int Arch Allergy Immunol (2008) 145:231–43. doi: 10.1159/000109292 PMC2576511 17914275

[125] Allan SE Crome SQ Crellin NK Passerini L Steiner TS Bacchetta R. Activation-induced foxp3 in human T effector cells does not suppress proliferation or cytokine production. Int Immunol (2007) 19(4):345–54. doi: 10.1093/intimm/dxm014 17329235

[126] Piccirillo CA Letterio JJ Thornton AM McHugh RS Mamura M Mizuhara H. Cd4(+)Cd25(+) regulatory T cells can mediate suppressor function in the absence of transforming growth factor beta1 production and responsiveness. J Exp Med (2002) 196(2):237–46. doi: 10.1084/jem.20020590 PMC2193919 12119348

[127] Tang Q Bluestone JA Kang SM. Cd4(+)Foxp3(+) regulatory T cell therapy in transplantation. J Mol Cell Biol (2012) 4(1):11–21. doi: 10.1093/jmcb/mjr047 22170955 PMC3695644

[128] Duggleby R Danby RD Madrigal JA Saudemont A. Clinical grade regulatory cd4(+) T cells (Tregs): moving toward cellular-based immunomodulatory therapies. Front Immunol (2018) 9:252. doi: 10.3389/fimmu.2018.00252 29487602 PMC5816789

[129] Mathew JM HV J LeFever A Konieczna I Stratton C He J. A phase I clinical trial with ex vivo expanded recipient regulatory T cells in living donor kidney transplants. Sci Rep (2018) 8(1):7428. doi: 10.1038/s41598-018-25574-7 29743501 PMC5943280

[130] Kazmi S Khan MA Shamma T Altuhami A Assiri AM Broering DC. Therapeutic nexus of T cell immunometabolism in improving transplantation immunotherapy. Int Immunopharmacol (2022) 106:108621. doi: 10.1016/j.intimp.2022.108621 35189469

[131] \Zhang Q Lu W Liang CL Chen Y Liu H Qiu F. Chimeric antigen receptor (Car) treg: A promising approach to inducing immunological tolerance. Front Immunol (2018) 9:2359. doi: 10.3389/fimmu.2018.02359 30369931 PMC6194362

[132] Mohseni YR Tung SL Dudreuilh C Lechler RI Fruhwirth GO Lombardi G. The future of regulatory T cell therapy: promises and challenges of implementing car technology. Front Immunol (2020) 11:1608. doi: 10.3389/fimmu.2020.01608 32793236 PMC7393941

[133] Lamarthee B Marchal A Charbonnier S Blein T Leon J Martin E. Transient mtor inhibition rescues 4-1bb car-tregs from tonic signal-induced dysfunction. Nat Commun (2021) 12(1):6446. doi: 10.1038/s41467-021-26844-1 34750385 PMC8575891

[134] Schwarz C Unger L Mahr B Aumayr K Regele H Farkas AM. The immunosuppressive effect of ctla4 immunoglobulin is dependent on regulatory t cells at low but not high doses. Am J Transplant (2016) 16(12):3404–15. doi: 10.1111/ajt.13872 27184870

[135] Govender L Wyss JC Kumar R Pascual M Golshayan D. Il-2-mediated in vivo expansion of regulatory T cells combined with cd154-cd40 co-stimulation blockade but not ctla-4 ig prolongs allograft survival in naive and sensitized mice. Front Immunol (2017) 8:421. doi: 10.3389/fimmu.2017.00421 28484450 PMC5399033

[136] Hall BM Hall RM Tran GT Robinson CM Wilcox PL Rakesh PK. Interleukin-5 (Il-5) therapy prevents allograft rejection by promoting cd4(+)Cd25(+) ts2 regulatory cells that are antigen-specific and express il-5 receptor. Front Immunol (2021) 12:714838. doi: 10.3389/fimmu.2021.714838 34912327 PMC8667344

[137] Ulbar F Villanova I Giancola R Baldoni S Guardalupi F Fabi B. Clinical-grade expanded regulatory T cells are enriched with highly suppressive cells producing il-10, granzyme B, and il-35. Biol Blood Marrow Transplant (2020) 26(12):2204–10. doi: 10.1016/j.bbmt.2020.08.034 32961369

[138] Kishimoto K Dong VM Issazadeh S Fedoseyeva EV Waaga AM Yamada A. The role of cd154-cd40 versus cd28-B7 costimulatory pathways in regulating allogeneic th1 and th2 responses in vivo. J Clin Invest (2000) 106(1):63–72. doi: 10.1172/JCI9586 10880049 PMC314364

[139] Shuai L Cheng Q Shen T Yi Z Wu X. Ctla4-ig abatacept ameliorates proteinuria by regulating circulating treg/il-17 in adriamycin-induced nephropathy rats. BioMed Res Int (2020) 2020:2347827. doi: 10.1155/2020/2347827 32420329 PMC7201454

[140] Cutolo M Soldano S Gotelli E Montagna P Campitiello R Paolino S. Ctla4-ig treatment induces M1-M2 shift in cultured monocyte-derived macrophages from healthy subjects and rheumatoid arthritis patients. Arthritis Res Ther (2021) 23(1):306. doi: 10.1186/s13075-021-02691-9 34952630 PMC8709961

[141] Badell IR La Muraglia GM Liu D 2nd Wagener ME Ding G Ford ML. Selective cd28 blockade results in superior inhibition of donor-specific T follicular helper cell and antibody responses relative to ctla4-ig. Am J Transplant (2018) 18(1):89–101. doi: 10.1111/ajt.14400 28637095 PMC5740006

[142] Khan MA Shamma T Altuhami A Ahmed HA Assiri AM Broering DC. Ctla4-ig mediated immunosuppression favors immunotolerance and restores graft in mouse airway transplants. Pharmacol Res (2022) 178:106147. doi: 10.1016/j.phrs.2022.106147 35227891

[143] Stenger EO Watkins B Rogowski K Chiang KY Haight A Leung K. Abatacept gvhd prophylaxis in unrelated hematopoietic cell transplantation for pediatric bone marrow failure. Blood Adv (2023) 7(10):2196–205. doi: 10.1182/bloodadvances.2022008545 PMC10196963 36724508

[144] Brandenburg S Takahashi T de la Rosa M Janke M Karsten G Muzzulini T. Il-2 induces in vivo suppression by cd4(+)Cd25(+)Foxp3(+) regulatory T cells. Eur J Immunol (2008) 38(6):1643–53. doi: 10.1002/eji.200737791 18493984

[145] Zorn E Nelson EA Mohseni M Porcheray F Kim H Litsa D. Il-2 regulates foxp3 expression in human cd4+Cd25+ Regulatory T cells through a stat-dependent mechanism and induces the expansion of these cells in vivo. Blood (2006) 108(5):1571–9. doi: 10.1182/blood-2006-02-004747 PMC1895505 16645171

[146] Wang S Gao X Shen G Wang W Li J Zhao J. Interleukin-10 deficiency impairs regulatory T cell-derived neuropilin-1 functions and promotes th1 and th17 immunity. Sci Rep (2016) 6:24249. doi: 10.1038/srep24249 27075020 PMC4831052

[147] Tran DQ. Tgf-beta: the sword, the wand, and the shield of foxp3(+) regulatory T cells. J Mol Cell Biol (2012) 4(1):29–37. doi: 10.1093/jmcb/mjr033 22158907

[148] Tran GT Hodgkinson SJ Carter NM Verma ND Plain KM Boyd R. Il-5 promotes induction of antigen-specific cd4+Cd25+ T regulatory cells that suppress autoimmunity. Blood (2012) 119(19):4441–50. doi: 10.1182/blood-2011-12-396101 22310911

[149] Schiering C Krausgruber T Chomka A Frohlich A Adelmann K Wohlfert EA. The alarmin il-33 promotes regulatory T-cell function in the intestine. Nature (2014) 513(7519):564–8. doi: 10.1038/nature13577 PMC4339042 25043027

[150] Do JS Visperas A Sanogo YO Bechtel JJ Dvorina N Kim S. An il-27/lag3 axis enhances foxp3+ Regulatory T cell-suppressive function and therapeutic efficacy. Mucosal Immunol (2016) 9(1):137–45. doi: 10.1038/mi.2015.45 PMC4662649 26013006

[151] Guo Y Mei Z Li D Banerjee A Khalil MA Burke A. Ischemia reperfusion injury facilitates lung allograft acceptance through il-33-mediated activation of donor-derived il-5 producing group 2 innate lymphoid cells. Am J Transplant (2022) 22(1963–75). doi: 10.1111/ajt.17084 PMC9357103 35510760

[152] Day AJ Milner CM. Tsg-6: A multifunctional protein with anti-inflammatory and tissue-protective properties. Matrix Biol (2019) 78-79:60–83. doi: 10.1016/j.matbio.2018.01.011 29362135

[153] Roura S Monguio-Tortajada M Munizaga-Larroude M Clos-Sansalvador M Franquesa M Rosell A. Potential of extracellular vesicle-associated tsg-6 from adipose mesenchymal stromal cells in traumatic brain injury. Int J Mol Sci (2020) 21(18):1–21. doi: 10.3390/ijms21186761 PMC7554813 32942629

[154] Gille I Claas FHJ Haasnoot GW Heemskerk MHM Heidt S. Chimeric antigen receptor (Car) regulatory T-cells in solid organ transplantation. Front Immunol (2022) 13:874157. doi: 10.3389/fimmu.2022.874157 35720402 PMC9204347

[155] Sicard A Lamarche C Speck M Wong M Rosado-Sanchez I Blois M. Donor-specific chimeric antigen receptor tregs limit rejection in naive but not sensitized allograft recipients. Am J Transplant (2020) 20(6):1562–73. doi: 10.1111/ajt.15787 31957209

